# The Philosophy of the Eye; Being a Familiar Exposition of Its Mechanism, and of the Phenomena of Vision, with a View to the Evidence of Design

**Published:** 1837-10

**Authors:** 


ART. VI.—*The Philosophy of the Eye; being a familiar Exposition of its Mechanism, and of the Phenomena of Vision, with a View to the Evidence of Design.* By JOHN WALKER, Lecturer in the Manchester School of Anatomy, &c.—*London,* 1837. 8vo. pp. 300; with numerous Woodcuts.

ALTHOUGH this work is in some degree calculated for the general reader, it will be highly useful to the medical student as an interesting and attractive introduction to the anatomy and physiology of the eye. Indeed it contains more information in both these departments than any single book which is likely to fall into the hands of practitioners generally, and we therefore recommend it to their notice. The plan of the treatise is good, the matter choice and well arranged, and every point of the least intricacy or difficulty is illustrated by excellent woodcuts. The constant references to design in the structure and functions of the wonderful organ of which it treats is none of its least recommendations as a work to be put into the hand of the younger members of our profession.

